# A scoping review of strategies for adolescents’ sexual and reproductive health role modelling

**DOI:** 10.4102/safp.v66i1.5859

**Published:** 2024-05-03

**Authors:** Tshiamo N. Ramalepa, Thinavhuyo R. Netangaheni

**Affiliations:** 1 Department of Nursing Science, School of Healthcare Sciences, Sefako Makgatho Health Sciences University, Tshwane, South Africa; 2 Department of Health Studies, College of Human Sciences, University of South Africa, Tshwane, South Africa

**Keywords:** adolescents, sexual and reproductive health, role modelling, adolescent role models, reproductive health

## Abstract

**Background:**

Individuals strive to maintain their sexual and reproductive health (SRH) by being exposed to correct information, having access to contraceptives, and promoting safe sex practices. Adolescent SRH promotion efforts should consider the influence of role models. This review explored the availability and nature of strategies and programmes on adolescents’ SRH role modelling and described them using a scoping review.

**Methods:**

Studies were retrieved from four databases and grey literature through a search of 223 studies. The databases included EBSCO-host, Medline, Sabinet, and Pubmed. Data extraction was guided by a data-extraction tool adapted from the JBI Manual for Evidence Synthesis. The characteristics of the selected studies were recorded in a Microsoft spreadsheet. Eleven studies published between 2014 and 2022 were included for the final review and analysed using thematic analysis.

**Results:**

Selected articles focused on adolescents’ SRH; however, only two studies focused particularly on role modelling. Nonetheless, some aspects of the findings and recommendations presented could be extrapolated to adolescents’ SRH role modelling. This includes adolescent–parent communication on SRH, community engagement, mentoring, positive role modelling, and information sharing through media campaigns.

**Conclusion:**

There is a lack of literature on SRH role modelling because most studies did not focus on role modelling as an aspect of SRH. Therefore, research needs to be conducted on strategies and programmes focusing on SRH modelling.

**Contribution:**

The findings of this scoping review may encourage the development and implementation of strategies and programmes targeting adolescents’ SRH throughout diverse communities to promote adolescent SRH.

## Introduction

Adolescents sexual and reproductive health (SRH) refers to a state of total well-being in all aspects of the reproductive system, including physical, mental, and social health of people between the ages of 10 and 19 years.^1,2^ The United Nations Population Fund (UNFPA) further explains that individuals strive to maintain their SRH by being exposed to correct information, having access to contraceptives, and promoting safe sex practices.^1^ For adolescents, maintenance of their SRH happens at a time when they are discovering and establishing their sexual and reproductive developmental changes. Sanci et al.^3^ suggested that because of these changes, adolescents recognise the connection between behaviour and consequences to a lesser extent, and may believe that they are invincible, leading to experimenting with drugs, and alcohol, as well as engaging in sexual activity early in life.

The World Health Organization (WHO) posits that SRH changes happen during a turbulent period in adolescents’ lives, where they are at risk of adolescent pregnancies and early parenthood, which perpetuates outcomes such as poverty and poor education attainment in adulthood.^4^ Likewise, during this time young people tend to take more risks, especially sexual risks, which can have serious implications such as unwanted pregnancies or sexually transmitted infections (STIs).^5^ Adolescent pregnancies have been a problem for developing and sub-Saharan countries; for instance, in 2021 it was estimated that 14% of adolescent girls had given birth before they turned 18 years globally.^6^ In the United States (US) it was reported that in 2020 around 15% of all live births among 15 to 19-year-olds were at least the mother’s second child.^7^

Undeveloped countries in Africa face more adversities related to adolescent SRH issues. In sub-Saharan Africa, child marriage, adolescent pregnancies, HIV transmission, and poor coverage of contraception are common, which puts adolescent SRH as a significant public health concern.^8^ Compared to countries such as the US and other European countries, adolescents in Africa encounter other adversities such as child marriages, sexual and gender-based violence, and female genital mutilation.^9^ This may be because of cultural and societal influences that impact the choices of adolescents of both genders. Cultural, social, and economic disparities in Africa perpetuate gender inequalities, which affect women’s ability to make their own SRH choices, which favour their health and well-being.^10^ Melesse et al.^8^ supported this notion by highlighting that the gender gap between male and female adolescents in Africa predisposes females to poor reproductive health outcomes because of early sexual debut and child marriages. Studies suggest that efforts to address SRH promotion focus mostly on young women, however; to properly address the SRH needs of adolescents, the role of young men must be emphasised.^11^ Sexual and reproductive health promotion efforts should also consider the influence of peers, parents, and members of the community, who may be regarded as role models for some adolescents.

Sexual and reproductive health role models refer to individual adolescents who look up to and aim to imitate their behaviours because they see it as desirable; such behaviour may be positive or negative.^12^ Price-Mitchel^13^ reiterated that role models are often seen as people who exhibit exceptional traits, such as the capacity to uplift others, communicate moral principles, and overcome challenges, however, they can have either a positive or negative influence on adolescents. Role models such as parents display characteristics that can influence adolescents’ behaviour, such behavioural influence depends on parent–child communication, the degree of closeness between them, monitoring and supervision, and transmission of values and knowledge.^14^ Munea et al.^15^ studied adolescent SRH from a sociocultural context. They asserted that sociocultural factors influence adolescents’ experiences, behaviour, and decision-making, such sociocultural factors are transmitted from the community and the society. Role modelling on the other hand plays a major role in how adolescents’ behaviour is influenced by these socio-cultural factors. Role models, as members of the community can be imitated by adolescents who may be regarded as impressionable. Furthermore, influences may also come from the communication styles and the content that is being communicated between adolescents and their role models.

In South Africa, adolescents’ SRH rights policies advocate for effective community supportive networks, quality health services, screening for STIs, counselling, and education for adolescents.^16^ The researcher has observed that there is a significant gap in the literature regarding the position of role models in SRH support and the influence of adolescents in the South African context. Literature on this phenomenon mostly originates from countries such as the US and some European countries.^11,17^ Sexual and reproductive health role modelling has not been explored in the South African context. Therefore, the study aimed to explore the availability of strategies and programmes on adolescents’ SRH role modelling using a scoping review.

## Objectives of the study

- To explore the availability of strategies and programmes on SRH role modelling using a scoping review and describe them using a scoping review
- To investigate the scope and quantity of literature on adolescents’ SRH role modelling

### Review questions

- What is the availability and nature of strategies and programmes on adolescents’ SRH role modelling?
- What is the scope and quantity of literature on adolescents’ SRH role modelling?

## Methodology

The study was conducted using a qualitative scoping review. A scoping review is a type of literature review that is used for evidence synthesis to systematically identify and map the availability of information on a particular topic or concept in a broader context.^18,19^ The scoping review in this study was conducted according to the methodological framework outlined by JBI by Peters et al.^20^ JBI Manual for Evidence Synthesis highlights that, unlike extensive systematic reviews, scoping reviews aim to provide an overview of the literature available on a certain topic.^21^ The JBI framework recommends that in a scoping review, the researcher must outline the review objective, questions, eligibility criteria, search strategy, evidence screening and selection, data extraction, and analysis of evidence, presentation of results and summary of evidence with the purpose of the review.^20^

### Eligibility criteria

Inclusion and exclusion criteria were formulated to refine and give relevant data and all criteria were applied to all search engines and databases. Studies that were eligible during the search included full journal articles, systematic reviews, official reports, dissertations, and theses, grey literature, studies that were published in English, studies that were published between 2013 and 2023, and both qualitative and quantitative research studies. Guidelines and policies were also consulted but there was no literature found. Studies that were not eligible for inclusion were articles that did not discuss adolescents’ SRH role modelling, abstracts, editorial, clinical reviews, and conceptual articles.

### Search strategy

The literature search included browsing through databases in search of articles that contain information on strategies and programmes for SRH role modelling. The search strategy included four databases, namely EBSCO-host, Medline, Sabinet, and Pubmed. With an aim to collect relevant strategies and programmes for SRH role modelling, the researcher consulted both national and international literature. The process recommended by the JBI Manual for Evidence Synthesis and the Preferred Reporting Items for Systematic Reviews and Meta-Aanalysis for Scoping Reviews (PRISMA-ScR) guidelines were used to select sources of evidence.^20,21^ A combination of keywords used for the search were ‘adolescent’, ‘sexual and reproductive health’, ‘role modelling’, ‘adolescent role modelling’ ‘sexual and reproductive role modelling’, and ‘role modelling strategies’. Because of the scarcity of sources on SRH role modelling and strategies, more combinations of keywords were used to broaden the search. Grey literature was also used to search for relevant articles, which resulted in five articles being found, however, the articles were not focused particularly on SRH role modelling. Other materials such as guidelines and policies were consulted online, yielding no results. A total of 223 identified sources were loaded into the Mendeley reference manager and converted into a RIS file, which was loaded into the Rayyan software program for screening and selection.

### Evidence screening and selection

After the search, all discovered citations were collected and imported into the Mendeley reference manager program. The references were then imported into the Rayyan software to manage and organise the selected sources for the scoping review and eliminate duplicates.^22^ The titles and abstracts were evaluated by the researcher and one reviewer who compared them to the researcher’s inclusion criteria. The full text of any potentially pertinent sources was obtained and their citation information was incorporated. The researcher and reviewer carefully evaluated the complete text of the chosen citations about the inclusion criteria. The scoping review noted and documented the reasons for excluding full-text sources of evidence that do not fit the inclusion criteria. At each level of the selection process, any discrepancies that arose between the researcher and the reviewers were handled through discussions.

### Charting the data

Data extraction or data charting summarises the results logically and descriptively, and it reveals the alignment between the results and the objectives or questions of the scoping review.^21^ The data that were extracted focused on literature about adolescents’ SRH role modelling. The data extraction or charting process was guided by a data-extraction tool adapted from the JBI Manual for Evidence Synthesis.^21^ The tool was used to extract details from the articles, such as authors, year of publication, source details, methods, and key findings that relate to the scoping review as reflected in Table 4. Data capturing was performed using a Microsoft Excel spreadsheet to record the counts, participants’ details, characteristics of data, methods, context, citation details, country, and the summary of findings related to adolescents’ SRH role modelling.

### Analysis of evidence

Data were analysed according to the three steps proposed in the framework by Arksey and O’Malley.^23^ The first step included an analytic framework using the PRISMA-ScR flow chart to provide an overview of the breadth of the literature regarding the strategies for SRH role modelling. The second step included a descriptive statistical analysis using tables to present the kind of studies selected. The descriptive statistics reported on types of publications of sources, year of publication, and the origin of the studies by country, are presented in Table 1, Table 2, and Table 3. In the third and last step, thematic analysis was used to select themes related to strategies and programmes focusing on adolescents’ SRH role modelling. The themes that emerged during the analysis were discussed in detail to reflect the description of SRH role modelling.

**TABLE 1:** Year of publication.

Year of publication	Number of studies (*n* = 11)	%
2020	3	27.2
2022	2	18.2
2015	2	18.2
2014	2	18.2
2021	1	9.1
2019	1	9.1

*Source:* Table 1: Years of publication:^39^ Mhlanga NL, Netangaheni TR. Risks of Type 2 diabetes among older people living with HIV: A scoping review. S Afr Fam Pract. 2023;65(1), a5623. https://doi.org/10.4102/safp.v65i1.5623

**TABLE 2:** Type of publication of sources.

Method	Number of studies (*n* = 11)	%
Qualitative	5	45.4
Mixed methods	2	18.2
Review	2	18.2
Theoretical article	1	9.1
Quantitative	1	9.1

*Source:* Table 2: Study design:^39^ Mhlanga NL, Netangaheni TR. Risks of Type 2 diabetes among older people living with HIV: A scoping review. S Afr Fam Pract. 2023;65(1), a5623. https://doi.org/10.4102/safp.v65i1.5623

**TABLE 3:** Origin of studies by country.

Country of origin	Number of studies (*n* = 11)	%
US	4	36.3
Ethiopia	2	18.2
Nigeria	1	9.1
Tanzania	1	9.1
Malawi	1	9.1
Cambodia	1	9.1
Chile	1	9.1

*Source:* Table 3: Place of origin of studies:^39^ Mhlanga NL, Netangaheni TR. Risks of Type 2 diabetes among older people living with HIV: A scoping review. S Afr Fam Pract. 2023;65(1), a5623. https://doi.org/10.4102/safp.v65i1.5623

US, United States.

### Ethical considerations

The research study was approved by the College Research Ethics Committee (CREC), College of Human Sciences, University of South Africa (Ref: Rec-240816-052).

## Results

**FIGURE 1:**
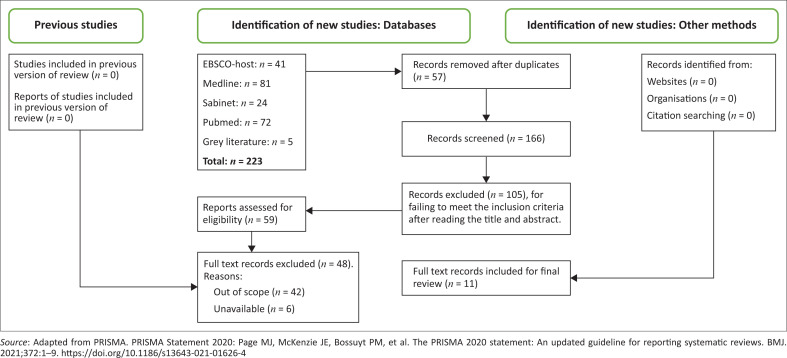
Preferred Reporting Items for Systematic Reviews and Meta-Analysis for Scoping Review flow chart for selection of studies.

Articles were retrieved from four databases and grey literature through a search of 223 studies. Fifty-seven duplicates were then removed, which resulted in 166 studies being available for abstract reading. The inclusion and exclusion criteria were applied to select relevant studies for the scoping review. A total 105 records were then excluded for failing to meet the inclusion criteria after reading the title and abstract, which left 59 records for full-text reading and eligibility. As there is little literature about SRH role modelling, 42 studies were excluded for being out of scope and focusing on adolescent SRH in general without role modelling strategies or programmes. Furthermore, six studies were unavailable for full-text reading. Lastly, 11 studies were then included for the final review. The process of selecting the studies is presented in Figure 1 adapted from the PRISMA flow chart.^24^

### Article summary characteristics

The characteristics of the selected articles are presented in Table 1, Table 2, Table 3, and Table 4. The 11 selected articles were published between 2012 and 2023. Table 1 presents the years of publication for the selected articles, five articles (*n* = 5) were published between 2020 and 2022, two studies (*n* = 2) between 2019 and 2021, and four studies (*n* = 4) between 2014 and 2015. The types of publication of sources in Table 2 indicates that most studies were qualitative (*n* = 5), then mixed method studies (*n* = 2) and review studies (*n* = 2) were two each. Lastly, there was only one theoretical article (*n* = 1) and one quantitative study (*n* = 1). Most studies focusing on SRH role modelling originate from the US (*n* = 4), two originate from Ethiopia (*n* = 2), and the rest originate from Nigeria (*n* = 1), Tanzania (*n* = 1), Malawi (*n* = 1), Cambodia (*n* = 1), and Chile (*n* = 1) (see Table 3).

**TABLE 4:** Summary of sources.

Author	Title	Method	The implication of SRH role modelling
Hailemariam et al. (2021)	Challenges faced by female out-of-school adolescents in accessing and utilising sexual and reproductive health service: A qualitative exploratory study in Southwest, Ethiopia	Qualitative	No specific strategies or programmes focus on SRH role modelling, however, some aspects of the article can be extrapolated. The article advocates for community-based awareness programmes, engaging community influences, and parents, and promotion of youth friendly services to address SRH of adolescents. Furthermore, the article encourages parent-adolescent communication and parental involvement in adolescents’ SRH programmes.
Mbachu et al. (2020)	Exploring issues in caregivers and parent communication of sexual and reproductive health matters with adolescents in Ebonyi state, Nigeria	Mixed methods	No specific strategies or programmes focus on SRH role modelling, however, some aspects of the article can be extrapolated. This article highlights the importance of parent–child communication: an event that rarely occurs. SRH content shared by parents consists of strict warnings and ambiguous information. Parents need to be reorientated to communicate better with adolescents. Early initiation of SRH communication was highlighted.
Wamoyi et al. (2014)	A review of interventions addressing structural drivers of adolescents’ sexual and reproductive health vulnerability in sub-Saharan Africa: implications for sexual health programming	Review	No specific strategies or programmes focus on SRH role modelling, however, some aspects of the article can be extrapolated. Most of the interventions addressed multiple structural factors, such as social norms, gender inequality, and poverty. Some interventions focused on reducing economic drivers that increased sexual risk behaviours. Others focused on changing social norms and thus sexual risk behaviours through communication. Social norms addressed included gender inequality, gender violence, and child socialisation. The interventions included components of comprehensive sexuality and behaviour change and communication and parenting.
Chimatiro et al. (2020)	The role of community leaders on adolescent HIV and sexual reproductive health and rights in Mulanje, Malawi	Qualitative	No specific strategies or programmes focus on SRH role modelling, however, some aspects of the article can be extrapolated. This article highlights that community leaders have many roles in adolescent HIV and SRH. These roles include advisory, encouragement, regulating and restricting cultural practices, formulating bylaws, and handling sexual abuse complaints. However, community leaders with religious affiliations have been shown to have different views from those representing other institutions not affiliated with religion.
Malango et al. (2022)	Parent–adolescent discussion on sexual and reproductive health issues and its associated factors among parents in Sawla town, Gofa zone, Ethiopia	Quantitative	No specific strategies or programmes focus on SRH role modelling, however, some aspects of the article can be extrapolated. The study highlights parent-to-adolescent discussion on reproductive health issues as the basis for a safe and healthy transition to adulthood. Continuous capacity building regarding SRH for parents is recommended to improve their knowledge support and increase the reach of the adult learning programme to decrease the level of illiteracy. Another recommendation would be to engage health providers with the tools and job aids to help them discuss the importance of parent–adolescent communication on SRH issues and include activities around this topic in donor-funded AYRH projects in Ethiopia.
Manlove et al. (2015)	Programmes to improve adolescent sexual and reproductive health in the US: A review of the evidence	Review	No specific strategies or programs focus on SRH role modelling, however, some aspects of the article can be extrapolated. Parent–youth relationship programmes and clinic-based programme evaluations more frequently showed impacts compared to other programme approaches with a particular focus on communication about sexual behaviour and romantic relationships.
Albertson et al. (2020)	Caregiver-endorsed strategies to improving sexual health outcomes among foster youth	Quantitative	No specific strategies or programmes focus on SRH role modelling, however, some aspects of the article can be extrapolated. (1) Strategies to communicate with youth about sexual health (trust-building, open/direct communication, tailoring information to each youth, creative conversation-starters, and self-education), (2) strategies for monitoring youth (e.g. tailoring monitoring to youth development/characteristics, clearly communicating rules, co-development of rules, spending time with youth/friends/friends’ parents, using technology, using a social support system), and (3) other strategies to promote positive youth development (advocating for youth, engaging youth in goal setting). Training that provides caregivers with (1) information on sexual health and normal adolescent development and (2) strategies and skills to help caregivers engage in open, evidence-informed communication, and monitor youth in a tailored, developmentally informed manner are likely to improve reproductive health and other outcomes among youth in foster care.
Kenny et al. (2019)	A qualitative exploration of the sexual and reproductive health knowledge of adolescent mothers from the indigenous population in Ratanak Kiri province, Cambodia	Qualitative	No specific strategies or programmes focus on SRH role modelling, however, some aspects of the article can be extrapolated. This study found that adolescents’ social interaction with family members and female villagers was the primary source of SRH information. Public health action must utilise this platform to increase SRH knowledge. Educating village elders and respected community members to lead community-based informal education programmes would contribute to increasing SRH knowledge and reducing misconceptions and inaccuracies prevalent in villages
Colarossi et al. (2014)	The Adult Roles Models Program: Feasibility, Acceptability, and Initial Outcomes	Mixed method	The study is about an adult programme for role models for adolescents’ SRH. The ARM programme is curriculum-based and led by trained parent–peer educators in community settings. The four-workshop weekly 2-h sessions were highly feasible and acceptable to the parents in our study, as evidenced by high attendance rates, satisfaction, and interest in the programme. Descriptive qualitative and quantitative data revealed high levels of acceptability and feasibility for parent participants. Participants travelled near and far by public transportation within the Bronx to attend the groups. Many participants noticed that our provision of food and cash incentives was helpful. Furthermore, the curriculum was conducted with a high level of fidelity and facilitator quality. Exploratory, small-scale RCT data show promising parent outcomes for frequency of communication, monitoring, and connectedness with children, and increases in knowledge about sexuality and adolescent development. We look forward to comparing youth reports related to the ARM intervention and conducting a larger-scale RCT in the future. Most analytic effects were interactions between group and time, such that as ARM intervention participants increased across outcomes, even if not as a significant main effect, control participants decreased. One explanation may be that the ARM intervention group parents practised skills over time and thus experienced more positive parent–child outcomes as their teens progressed through developmental stages that may have created more challenges for control group parents. This research adds to the small, but growing, literature on the importance of parent sexuality education and its impact on adolescent sexual and reproductive health. We present a new curriculum model that is highly feasible and acceptable and can be performed with fidelity. Results demonstrate promising parent outcomes for frequency of communication, monitoring, and connectedness with their children, and increases in knowledge about sexuality and adolescent development.
Svanemyr et al. (2015)	Creating an enabling environment for adolescent sexual and reproductive health: A framework and promising approaches	Review	This study provides relevant strategies that can be adopted and used as SRH role modelling strategies. The strategies include Creating safe spaces for adolescent girls, parental engagement, mentoring, and positive role modelling, mobilisation of adults and community leaders, working with boys and encouraging men to promote gender-equitable norms, Media campaigns, and large-scale communication programmes, and promoting laws and policies and their implementation.
Obach et al. (2022)	Strengths and challenges of a school-based sexual and reproductive health programme for adolescents in Chile	Qualitative	No specific strategies or programmes focus on SRH role modelling, however, some aspects of the article can be extrapolated. Permanent and expedited student access to sexual and reproductive healthcare is achieved, and affectional bonds are developed between students and the program’s health staff. The programme assists female participants in imagining and forming identities that are not inherently tied to motherhood. It also assists boys and LGBTQ+ adolescents in feeling included as relevant actors in sexual and reproductive health and decision-making. According to participants, the affectivity and sexuality component of the programme encourages self-care and responsibility in sexual and emotional relationships. Effective dialogue, communicating one’s feelings, and caring for the feelings of others, are the focus of the programme. 3A it also encourages encourages reflection on gender stereotypes and norms regarding sexuality.

*Source:* Adapted from the JBI Manual for Evidence Synthesis 2023: Francis E. JBI manual for evidence synthesis [homepage on the Internet]. 2022 [cited 2023 Apr 14]. Available from: https://jbi-global-wiki.refined.site/space/MANUAL/4687770/11.3+The+scoping+review+and+summary+of+the+evidence

SRH, sexual and reproductive health.

Table 4 presents a summary of selected studies based on the authors, title of the article, research methods used, and implications of SRH role modelling. All the articles selected focused on adolescents’ SRH, however, only one article focused particularly on role modelling. Nine studies had no specific strategies or programmes focusing on SRH role modelling. Nonetheless, some aspects of the findings and recommendations presented could be extrapolated to adolescents’ SRH role modelling. This includes parental involvement and communication, community-based programmes, engagement of health providers, youth friendly services, information provision and health education, and involvement of peer educators.

Two studies by Colarossi et al.^17^ and Svanemyr et al.^25^ present strategies that focus on SRH role modelling. The strategies include a curriculum-based peer educator SRH role modelling programme, creating safe spaces for adolescent girls, parental engagement, mentoring, and positive role modelling, Mobilisation of adults and community leaders, working with boys, and encouraging men to promote gender-equitable norms. Other strategies focused on the use of media campaigns and large-scale communication programmes and promoting and implementing laws and policies on SRH role modelling.

### Emerging themes from the selected studies

The selected studies yielded five themes: adolescent–parent communication on SRH, community engagement, mentoring, positive role modelling, and information sharing through media campaigns. The themes identified were extrapolated to describe strategies applicable to SRH role modelling. However, the themes should not be conflated with SRH role modelling.

#### Theme 1: Adolescent–parent communication on sexual and reproductive health

Adolescent parent communication is the most common strategy for adolescent SRH role modelling found in the selected studies. Parent–adolescent communication and parental involvement were proposed as some of the strategies included in SRH programmes.^26^ Such strategies promote and advocate for a community-based awareness approach in addressing SRH issues among adolescents. This was supported by another article that shared the same sentiments about adolescent–parent communication as an impactful strategy to address adolescent SRH. Parents use their knowledge and experience to guide adolescents in making the right decision and they usually occupy the role model status.^27^

Sexual and reproductive health programmes focusing on parent–youth relationships encourage parents to communicate with their adolescents about sexual behaviour and romantic relationships, which may be an effective strategy for SRH role modelling.^27^ Furthermore, it was recommended that funded SRH projects in Ethiopia focus on parental engagement and adolescent–parent communication in SRH issues, as well as the facilitation of activities around this topic.^28^ Despite numerous studies advocating for adolescent–parent communication as an effective strategy to improve adolescent SRH, there are also challenges identified in applying the strategy. The main difficulty impeding the effectiveness of this strategy is the content shared by parents with their adolescents.^29^ The content shared by parents contains strict warning messages to adolescents and the advice given during such conversations is often unclear.^29^ Parents need to acknowledge their position as important role models to their adolescents. Moreover, parents must be continuously capacitated to improve their knowledge to communicate effectively with their children.^28^ The importance of the early beginning of SRH communication between adolescents and parents should be emphasised.^29^

#### Theme 2: Community engagement

Community engagement is a strategy that can be applied in SRH role modelling because community members play a critical role in influencing the health choices of adolescents. Community-based SRH programmes are important to create awareness and educate community members to be proficient members when it comes to the lives of adolescents.^26,30^ This applied to role modelling because educating community members and respected community people to lead community-based informal education initiatives would help to increase SRH understanding while minimising common misconceptions and inaccuracies in the community.^30^ Furthermore, role models such as family members and female community members were regarded as the source of information for female adolescents when it came to SRH.^30^ There are roles that community members occupy in promoting adolescents’ SRH, these roles include guidance, encouraging, regulating, and banning cultural customs, drafting ordinances, and addressing sexual abuse complaints.^31^ Studies that supported this notion affirmed that community leaders are responsible for teaching adolescents about social issues such as social norms, gender-equitable norms, and sexual behaviour.^25,32^ Moreover, community religious leaders play an important role in imparting knowledge and information from a religious narrative.^31^

#### Theme 3: Mentoring and positive role modelling

Mentoring and positive role modelling are essential in ensuring that adolescents imitate positive SRH choices in their daily lives. Creating safe spaces for adolescent girls, parental participation, mentoring, positive role modelling, mobilisation of adults and community leaders, engaging with boys, and motivating males to promote gender-equitable norms were among some of the strategies identified.^25^ Community members such as parents, religious leaders, teachers, siblings, relatives, and healthcare workers can all act as positive role models in the community. School-based SRH programme for adolescents in Chile promoted permanent and expedited access to SRH care, and affectionate bonds formed between students and the programme’s health personnel.^33^ This implies that through the bonds, positive role modelling can emerge between adolescents and health personnel. Adolescents in the school-based programme were allowed to improve their SRH by learning about SRH decision-making, self-care, and being responsible in sexual and emotional relationships.^33^

A study by Colarossi et al.^17^ reported on an adult program for role models for adolescents’ SRH. The curriculum-based programme is a 4-week workshop led by parent-peer educators in the community.^17^ Data from the study suggest that encouraging parent outcomes in terms of frequency of communication, monitoring, and connectivity with children improves knowledge about sexuality and adolescent development.^17^ A study focusing on caregiver-endorsed strategies to improve SRH outcomes among youth suggested several strategies for positive role modelling and mentorship.^34^ The strategies include communicating with youth about sexual health, monitoring youth, and other positive youth development, training that teaches caregivers about sexual health and normal adolescent development, as well as strategies and skills to help caregivers engage in open, evidence-informed communication and monitoring of adolescents.^34^

#### Theme 4: Information sharing, media campaigns

Information sharing regarding SRH can come in various ways, including the use of media as a tool to reach many people. Continuous capacity building can improve the SRH knowledge of potential role models such as parents.^28^ Knowledge improvement is imperative because it means that role models can then impart accurate, relevant, and clear information to adolescents.^28^ This is supported by Albertson et al.^33^ who reiterated that training teaches parents about sexual health and normal adolescent development and improves their skills to help them engage in open, evidence-informed communication with adolescents, which improves their reproductive health outcomes. Recommendations to improve information sharing regarding adolescents SRH, included promoting laws and policies, as well as their implementation, through media campaigns and large-scale communication programmes.^25^

## Discussion

This scoping review mapped out the availability of literature and strategies on adolescents’ SRH role modelling. The results of the scoping review indicate that there is an unavailability of literature focusing on SRH role modelling. However, there are a few studies that contain strategies for SRH role modelling, and such studies focus on adolescents’ SRH in general. The four themes identified focus on different aspects of addressing SRH role modelling in communities. However, within each theme, communication seems to be the most common strategy to address adolescent SRH. In addition, the themes suggest that the link between role models in the community and adolescents stems from the notion that adolescents often lack knowledge regarding SRH issues.

Adolescent–parent communication is one of the most common strategies to address SRH modelling. The parental bond with their adolescent has a significant role in paving the route for them, both parent and adolescent must have a favourable level of understanding and progress towards a more approachable connection.^35^ It is important to involve parents and caregivers as crucial partners in nurturing and supporting healthy adolescent development, particularly when it comes to issues concerning their SRH choices.^36^ Parents in some instances assume the role model status because they are close to adolescents and they spend most of their time with them at home. Therefore, parents become the primary source of information, making the content that they share with adolescents very important.

Parents and caregivers assume role model status when they engage in positive conversations and offer guidance to adolescents so that they can make informed SRH choices. Furthermore, Kapetanovic et al. posit that parent–adolescent communication can provide a comfortable atmosphere for adolescents to willingly speak with their parents about their daily activities and life plans. However, it is not always easy for parents to communicate with adolescents about their daily activities and SRH because parents do not know what to say in some cases.^29^ Conversely, when adolescents provide information about their whereabouts and activities, parents have more possibilities to guide and encourage them to make the right choices.^37^

Parents and caregivers remain the primary people who offer guidance and support to adolescents, however, the whole community must be involved in addressing the SRH of adolescents. In Zambia, using community-led strategies to provide information on the responsiveness to adolescents’ SRH and promoting adolescent access to SRH information improved the lowering of early pregnancy and marriage rates, as well as maternal, neonatal, and child health.^38^ These community-based strategies to promote SRH role modelling can be fostered by community-based role models such as parents, teachers, religious leaders, and healthcare providers. These community members are highly regarded by adolescents and can help shape their SRH behaviour by providing health information to them.^31^ Furthermore, community leaders in Malawi were reported to have contributed to the reduction of maternal mortality in Malawi because they make decisions and govern cultural practices and beliefs in their communities.^31^ The choices made by community leaders directly impact the actions of adolescents because of the influence related to the cultural practices, cultural norms, and values in communities. Information sharing between adolescents and community role models may be hindered by the lack of role-modelling-focused initiatives in the community. Moreover, community-based role models such as parents, teachers, and community leaders may lack the relevant resources to facilitate different information-sharing and media campaigns focusing on SRH health content. This may suggest that members of the community also need to be capacitated so that they are able to play their SRH role modelling role.

## Conclusion

The scoping review aimed to explore the availability of strategies and programmes on adolescents’ SRH role modelling and describe them using a scoping review. The scoping review revealed that there is a lack of literature on SRH role modelling because most of the studies found did not focus on role modelling as an aspect of SRH. However, the findings and recommendations from the selected studies highlight strategies that can be extrapolated and used in addressing SRH role-modelling efforts in communities. The extrapolations only from the themes initiate a conversation regarding strategies for adolescents’ SRH role modelling and should not be conflated. The findings of the scoping review revealed common strategies and programmes that can be used for SRH role modelling, this includes programmes and strategies focusing on adolescent–parent communication on SRH, community engagement, mentoring, positive role modelling, and information sharing through media campaigns. Furthermore, this review highlights the gap in research and literature regarding SRH role modelling. Therefore, it is recommended that more research needs to be conducted on strategies and programmes focusing on SRH modelling. Strategies and programmes targeting adolescents’ SRH role modelling need to be developed and implemented throughout diverse communities to promote adolescent SRH. Moreover, communication strategies and programmes could provide opportunities to promote and cultivate SRH role modelling.

### Limitation

The following are the limitations of this review:

- ‘Sexual and reproductive health role modelling’ is a relatively new concept, therefore, there was only one study that focused particularly on this concept. Other studies included concepts that can be applied to SRH role modelling.
- This review included only four databases, however, there are many more databases available. Using four databases may have led to a selection bias in this study.
- This scoping review did not include a full appraisal of the selected studies, which limited the extent of the argument the selected studies contribute to strategies on SRH role modelling.
- The identified strategies were not tested for effectiveness and applicability.
- Because of the scarcity of studies, this is a limited scoping review.
